# Objective Job Demands of Oneself and One’s Partner, and Depressive Symptoms. Evidence from a Nationally Representative Longitudinal Study

**DOI:** 10.3390/ijerph182312688

**Published:** 2021-12-01

**Authors:** Benedikt Kretzler, Hans-Helmut König, André Hajek

**Affiliations:** Department of Health Economics and Health Services Research, University Medical Center Hamburg-Eppendorf, Hamburg Center for Health Economics, 20246 Hamburg, Germany; h.koenig@uke.de (H.-H.K.); a.hajek@uke.de (A.H.)

**Keywords:** job demands, spillover, mental health, partnership, depressive symptoms, depression, stress

## Abstract

Background: Job characteristics are an important predictor of depressive symptoms. Recent research detected unemployment’s spillover effects on spouses’ depressive symptoms, but there is still a lack of studies that examine the association between objective job demands of oneself and one’s partner and depressive symptoms. Methods: Data were derived from the German Ageing Survey (DEAS), which is a representative sample that includes individuals aged 40 years and older. Psycho-social, physical, and overall job characteristics were assessed objectively, using a validated index developed by Kroll. Depressive symptoms were quantified by the Center for Epidemiologic Studies Depression Scale (CES-D). Results: Regarding fixed-effects regression, we found no significant association between the own or the partner’s job demands and depression among the total sample and among men. However, among women, both increasing psychosocial demands of one’s own occupation and physical job demands of one’s partner’s occupation were related to higher levels of depression, as well as the partner’s overall job demands. Conclusions: The findings of the present longitudinal study highlight the association between job demands and depressive symptoms in women, but not in men, especially regarding the partner’s employment characteristics. Efforts to reduce the burden of high job demands may be helpful. This could help alleviate depressive symptoms. In turn, geriatric giants caused by increased depressive symptoms, such as frailty, could be postponed.

## 1. Introduction

Depressive symptoms are a common mental health problem, characterized by sadness, tiredness, and loss of interest [1]. Worldwide, 264 million people are affected. Depressive symptoms are a serious threat to health [2] and quality of life [3]. They tend to increase the risk of frailty [4], morbidity [5], and mortality [6], and decrease the health-related quality of life [7]. Moreover, they cause a tremendous economic burden. For example, in the European Union, their annual costs are about 118 billion euros, or 253 euros per inhabitant [8].

Due to this, efforts have been made to explore the determinants of depressive symptoms. For example, Piso, Mayatake, and Thase found out that developmental factors, such as an early childhood trauma or a poor relationship with the caretaker, predict depressive symptoms in later life [9]. They also revealed certain personality traits, particularly neuroticism and chronic stress, as correlates of depressive symptoms. Furthermore, having depressive people in one’s social network can increase the severity of one’s own symptoms as well [10]. Other risk factors are a younger age and lack of control [11], missing social support, and, particularly among older adults, functional decline [12].

Though some of these variables, such as stress and lack of control, are already related to several workplace settings, there are also many studies that investigated the association between several job strain dimensions and depression: Depressive symptoms may be caused by stress [13], as well as overcommitment and imbalance [14]. Low decision latitude and high job insecurity were found to increase the prevalence of depression [15,16], as dangerous working conditions and flexible working hours did [17]. On the other hand, optimism [18] and job satisfaction [19] were shown to alleviate depressive symptoms.

Longitudinal studies and meta-analyses also found a positive relationship between job strain and depression: high job demands increase the probability of depressive disorders [20,21,22], and may even cause burn-out [23].

In the related field of research on the effects of unemployment, Marcus found that the effect of exogenous unemployment on an individual’s mental health is as high as that of its partner [24]. Furthermore, an involuntary job loss was shown to reduce a female spouse’s mental health in case of subsequent long-term unemployment [25]. A similar pattern applies on life satisfaction [26] and subjective well-being [27].

Although there are many studies that have focused on the association between job characteristics and depressive symptoms, previous studies did not provide much evidence about spillover effects (i.e., externalities of depression predictors, such as job demands, on third persons) on the partner. To the best of our knowledge, no other study has yet investigated the effect of job demands on the spouse’s depressive symptoms.

To fill this research gap, the aim of our study was to investigate the relationship between objective job demands (of oneself and one’s partner) and one’s own depressive symptoms based on data from a nationally representative longitudinal study of middle-aged and older adults.

## 2. Materials and Methods

### 2.1. Sample

We used longitudinal data from the German Ageing Survey (DEAS), funded by the Federal Ministry for Family Affairs, Senior Citizens, Women and Youth in Germany. This is a nationally representative, prospective cohort study of community-dwelling Germans aged 40 and older. Data were collected through a standardized questionnaire carried out by trained interviewers.

The first survey of the ongoing DEAS occurred in 1996 and was followed by five waves in 2002 (wave 2), 2008 (wave 3), 2011 (wave 4), 2014 (wave 5), and 2017 (wave 6). As our instrument for assessing job demands was only included in 2002 and 2008, we exclusively used data from the second and the third wave in our current study. Usually, all follow-ups cover both participants who had already been interviewed and new participants who were interviewed for the first time—except for the fourth and sixth waves, which only included individuals who already had participated before. During the second wave, 5194 subjects were interviewed, and during the third wave, data were obtained from 8200 individuals. In 2008, the response rate for new participants was 35.7%. In the ongoing study, the retention rate, which is the share of individuals in the sample that have already been interviewed before, increased from 28.1% in 2002 to 32.4% in 2008. For further information, please see the reports on data and methods of the DEAS [28,29].

Out of all participants in the second and third wave, only those who were living in any kind of partnership were included in our study. Furthermore, the sample that provides data concerning the partner’s job demands (*n* = 2518) is smaller than the one with values for the own job demands (*n* = 3229). Despite that, there are also some cases where only data for the partner’s and not for the own job demands were reported (*n* = 621). Thus, our descriptive results describe the characteristics of all individuals that were included in at least one of the two regression analyses we carried out. Our final analytical sample size was *n* = 3850.

Written informed consent was given by all participants.

### 2.2. Dependent Variable

Depressive symptoms were assessed by the Center for Epidemiologic Studies Depression Scale (CES-D) [30], a widely used tool whose 15 items generate a score from 0 (no depressive symptoms) to 45 (severe depressive symptoms). In this study, Cronbach’s Alpha was 0.88. The CES-D proved to have adequate psychometric characteristics [31].

### 2.3. Independent Variables

The Kroll index [32] was used to quantify job demands, our main variable of interest. This instrument was developed to provide a comprehensive tool that should be applied in studies that do not particularly focus on job issues, but nevertheless aim to contain a valid item to quantify job strain. It is composed of 39 items, which are allocated to two categories of job demands: 13 of the 39 items measure physical job strain (e.g., carrying heavy objects or performing noisy work), and the remaining 26 items quantify psychosocial job strain (e.g., support by coworkers or multi-tasking work). All 39 items together generate the value for overall job demands. Every item has a range from 0 to 10. Its score depends on the occupation to which it was allocated: for every occupation according to the classification of the International Labour Organization [33], the specific item score was pre-calculated. After that, the scores were standardized across all occupations. Consequently, the score represents the decile of job demands, respective to all professions. For example, a score of 1 means that the job demands of an occupation are in the lowest decile. The Kroll index is validated [32] and has been used in some previous studies [34,35,36].

In regression analysis, it was adjusted for several covariates. Our regression model includes age, marital status (reference: married, living together with spouse), self-rated health, rated on a five-point scale from 1 (very good) to 5 (very bad), and the number of important people in one’s social network (from 0 to 9). Furthermore, we included physical functioning assessed by the correspondent subscale of the SF-36 [37]. It ranges from 0 to 100, with 0 as the worst and 100 as the best physical functioning.

### 2.4. Statistical Analysis

Estimates from cross-sectional observational studies can produce inconsistent estimates (due to time-constant unobserved heterogeneity). That issue can be tackled by panel regression models [38]: based on the assumption of correlation between time-constant factors (e.g., genetic disposition) and regressors, those models can be divided into different approaches. In our study, we assumed that time-constant factors and regressors are correlated. Thus, random effects regression would produce inconsistent estimates, as its key assumption is violated. Unlike this, fixed effects (FE) regression still provides consistent results—even when such a correlation is present [39]. Therefore, we favored FE regressions. This choice was also supported by Sargan–Hansen tests (e.g., for physical job demands as key explanatory variable: Sargan–Hansen-statistic = 110.063, *p* < 0.001).

The standard linear FE estimators are within-estimators. Hence, they only consider the variations within individuals over time. As depressive symptoms and overall job demands can vary within individuals over time and therefore meet the necessary criteria, they can be examined using FE regression analysis. However, this also means that the main effects of time-constant factors (such as sex or country of origin) are not estimated in FE regressions. On the other hand, it should be noted that time-constant factors (unobserved and observed) are implicitly controlled in FE regressions and do not bias FE estimates [39].

The FE estimator solely relies on data from those whose objective job demands changed over the sample period (i.e., from 2002 to 2008). Thus, it corresponds to an average treatment effect of the treated. As Brüderl and Ludwig pointed out, this is not a weakness of the FE approach [38]; it simply reflects the fact that only some older individuals had changes in job demands and depressive symptoms.

## 3. Results

### 3.1. Sample Characteristics

Table 1 shows socio-demographic characteristics for the total (analytical) sample and stratified by gender. Of the 3850 individuals, 49.93% were female. Mean age was 50.71 years (SD: 6.27), and age range was from 40 to 75 years (with n = 11 individuals (0.18%) being 67 years or older, which is the retirement age in Germany). In total, 76.03% of the respondents were married and living together with their spouse. The average self-rated health was 2.21 (SD: 0.77). The average physical functioning score was 93.61 (SD: 12.63). The average number of important people in regular contact was 4.91 (SD: 2.77).

Mean physical job demands were 5.16 (SD: 2.75), mean psychosocial job demands were 5.49 (SD: 2.98), and mean overall job demands were 5.22 (SD: 2.84). Mean partner’s physical job strain was 5.13 (SD: 2.79), mean partner’s psychosocial job strain was 5.35 (SD: 2.99), and mean partner’s overall job strain was 5.16 (SD: 2.88).

### 3.2. Regression Analysis

The findings of the FE regression analysis are displayed in Table 2 (with one’s own job demands as the main independent variable) and Table 3 (with job demands of one’s partner as the main independent variable).

FE regression revealed that changes in psychosocial job demands are associated with increases in depressive symptoms among women (ß = 1.09, *p* < 0.01; Table 2). Among the job demands of oneself, no other significant associations were found.

Regarding control variables, decreases in depressive symptoms were associated with increases in age (total sample; men). The end of a marriage was associated with an increase in depressive symptoms among men, whereas it significantly decreased depressive symptoms among women. A worsening self-rated health was associated with increases in depressive symptoms among men. Increases in the number of important people in regular contact are associated with increases in depressive symptoms among women.

Regarding job demands of the individual’s partner (Table 3), increases in physical job demands (ß = 1.48, *p* < 0.001) and overall job demands (ß = 0.94, *p* < 0.01) were associated with increases in depressive symptoms among women. In addition, increases in physical job demands of one’s partner were marginally significantly associated with increases in depressive symptoms among the total sample (ß = 0.61, *p* < 0.10). No other significant relationships were revealed regarding the key independent variables.

With regard to covariates, increases in age were associated with decreases in depressive symptoms in the total sample and stratified by sex. A worsening self-rated health was associated with increases in depressive symptoms in the total sample and in women. Increases in physical functioning were associated with decreases in depressive symptoms in the total sample and in women. An increase in the number of important people in regular contact was associated with an increase in depressive symptoms among the total sample.

## 4. Discussion

### 4.1. Main Findings

Based on a large nationally representative sample, the aim of this study was to examine the link between (objective) job demands (of an individual itself and of the individual’s partner) and depressive symptoms using a longitudinal approach.

Linear FE regressions revealed that increasing psychosocial job demands lead to higher depressive symptoms among women. Moreover, the partner’s physical and overall job demands also increased depressive symptoms among women.

### 4.2. Relation to Previous Research and Possible Explanations

The key finding of our study that increased job demands do not only increase depressive symptoms for individuals themselves, but also for their partners, seems well-grounded, as previous research stated both a positive association between job demands and depression [16,20,22] and the vulnerability of one’s own depression towards one’s partner’s occupational factors [24]. Our hypothesis, which was that increasing job demands of an individual might also increase its partner’s depressive symptoms, was partly confirmed. This finding add to our current knowledge. 

However, regarding the differentiation between physical and psychosocial job demands, there are some results of our study that are not supported by previous studies. Two systematic reviews identified several psychosocial job components, such as low decision latitude, to be associated with depressive symptoms [16], maybe particularly among men [40]—in contrast to our study, where women were more affected by psychosocial job demands. In between is Roxburgh, who detected that the effects do not significantly differ among both sexes, but that women are generally more exposed to psychosocial job demands [41]. In terms of the physical job demands, there is also some other evidence. Investigating a sample of Iranian nurses, Bagheri Hossein Abadi et al. revealed a significant positive relationship between physical job demands and depression [42], which was not found in our study. However, it should be noted that there are major differences between the samples of both examinations (e.g., Bagheri Hossein Abadi et al. examined 730 Iranian nurses, whereas we included community-dwelling individuals aged 40+ in Germany).

Regarding the psychosocial job demands, the positive association with depression that we detected might be explained by the positive relationship between several job demands-related factors such as stress [13] or imbalance [14] to depression [43]. Its sole occurrence among women might be caused by the higher vulnerability of the female gender towards depressive symptoms, particularly among middle-aged [44] and elderly [45] populations, of which our sample was made up. A corresponding pathway seems reasonable with respect to the association between the partner’s job demands and one’s own depressive symptoms. Studies have shown that physical work can increase the stress [46] and damage the mental health [47] of an individual, and that such developments can lead to increased depressive symptoms for the individual’s partner, more particularly among women [10,48].

Thus, there are other pathways that seem to be quite reasonable. For example, the increase in the physical job demands could also indicate a change from a white-collar (performing desk work) to a blue-collar (performing manual work) employment. The first one tends to claim a higher prestige for itself [49]. Considering that the DEAS only includes individuals aged 40 years and older, contemporary ideas of gender equity and both partners being employed might not be as present as in the Millennials generation [50], and particularly women might define themselves at least partially through their partner’s employment. Hence, they might suffer from the social descent of their husband, which could explain our findings as well.

Finally, referring to the utilization of an objective measure to quantify job demands, our results partly differed from those of other previous studies where the participants rated their job strain themselves. Two recent studies did not reveal a predictive effect of job demands on depression overtime in their fully adjusted models [51,52], but some older studies detected an increased likelihood of depressive symptoms that is caused by specific job traits [13,14,15,17]. A possible explanation might be the existence of bidirectional relations between job demands and depression, e.g., that the decrease in productivity that comes along with depressive symptoms [53] might also increase one’s (subjective) job demands. Regarding this, our findings also correspond to those of Åhlin, LaMontagne and Magnusson Hanson [51], who were the first to assess the relation between depressive symptoms and subsequent work characteristics and to control for time-invariant factors but used a subjective indicator of job demands. They revealed that work efforts are positively associated with depressive symptoms both in the short and in the long term.

### 4.3. Strenghts and Limitations

Our study relies on a large and nationally representative sample of German adults in their second half of life. The instruments used to quantify our key variables (objective job demands and depressive symptoms) were valid and reliable. In addition, this is one of only a few studies that provide a longitudinal procedure with objective measures. This means that many possible biases are eliminated: First, the statistical analysis controls for time-constant unobserved heterogeneity, which is a serious threat for cross-sectional approaches. Second, longitudinal studies are indicative of causal effects [54]. Third, the objective Kroll index, which is based on the classification of the International Labour Organization, provides valid estimates for job demands that are not biased by any subjective influences. Moreover, to the best of our knowledge, this is the first study investigating the influence of job demands on depressive symptoms in the context of partnership. It is embedded in a growing body of literature on the effect of unemployment, which revised perceptions of the costs of job loss [24].

Nevertheless, our study also has several weaknesses. The German Ageing Survey has a small sample selection bias, as elderly persons were oversampled to ensure a reasonable number of individuals in various demographic subgroups [29]. That may slightly affect the representativeness of our sample. Moreover, we cannot fully preclude reverse causality in the association between job demands and depressive symptoms: for example, depressive symptoms may reduce one’s job performance, which may lead to being forced into a lower paid job [55]. However, strategies to overcome endogeneity (such as instrumental variable approaches) heavily rely on strong instruments. In the case of weak instruments, these approaches would produce strongly biased estimates. Therefore, we used FE regressions in our study. Finally, it should be mentioned that our data were obtained through self-reports. This can also be considered as a limitation, because individuals’ answers may be influenced by other, unknown factors that one cannot control for in the statistical analysis. On the other hand, the instruments that were used, especially the CES-D, were shown to be valid and reliable [56].

## 5. Conclusions

Among women, we found increases in depressive symptoms to be associated with increases in psychosocial job demands, and with increases in partner’s physical and overall job demands. This might have several implications for the prevention of depression or the weakening of its negative impacts through the reduction of job stress/demands. On the other hand, medical directors and physicians should also keep tabs on the spouses or partners of highly strained individuals, as particularly women are in danger of developing depressive symptoms due to increasing partner’s job demands. Through this, depressive symptoms in older age could be alleviated. Consequently, the economic burden associated with increased depressive symptoms in older age could be reduced. Moreover, other geriatric giants caused by increased depressive symptoms, such as frailty, could be postponed. Finally, future research that regards the association between job demands and depressive symptoms and considers spillover effects on third parties is required to further explore the underlying mechanisms.

## Figures and Tables

**Table 1:** Sample characteristics for the individuals included in the regression analysis, stratified by sex.

	Total Sample		Men		Women	
	N/Mean	%/SD	N/Mean	%/SD	N/Mean	%/SD
Age	50.71	6.27	50.97	6.42	50.43	6.08
Marital status:						
Married, living together with spouse	2927	76.03%	1554	78.13%	1373	73.78%
Other	923	23.97%	435	21.87%	488	26.22%
Self-rated health (from 1 = very good to 5 = very bad)	2.21	0.77	2.21	0.76	2.21	0.77
Physical functioning (from 0 = very low to 100 = very high)	93.61	12.63	94.57	11.75	92.58	13.43
Number of important people in regular contact	4.91	2.77	4.68	2.76	5.15	2.76
Physical job demands	5.16	2.75	5.36	2.95	4.94	2.50
Psycho-social job demands	5.49	2.98	5.61	2.85	5.36	3.11
Overall job demands	5.22	2.84	5.46	2.79	4.96	2.87
Physical job demands (partner)	5.13	2.79	4.80	2.51	5.46	3.01
Psycho-social job demands (partner)	5.35	2.99	5.16	3.16	5.54	2.80
Overall job demands (partner)	5.16	2.88	4.78	2.89	5.54	2.82
Depressive symptoms (from 0 = no depressive symptoms to 45 = severe depressive symptoms)	5.91	5.63	5.46	5.16	6.40	6.07
Number of individuals	3850	100.00%	1989	51.66%	1861	48.34%

**Table 2:** Determinants of depressive symptoms (total sample and stratified by sex)—*with own objective job demands* (1: physical job demands, 2: psycho-social job demands; 3: overall job demands) as main independent variable. Results of multiple linear FE regressions.

	(1.1) Depressive Symptoms—Total Sample	(1.2) Depressive Symptoms—Men	(1.3) Depressive Symptoms—Women	(2.1) Depressive Symptoms—Total Sample	(2.2) Depressive Symptoms—Men	(2.3) Depressive Symptoms—Women	(3.1) Depressive Symptoms—Total Sample	(3.2) Depressive Symptoms—Men	(3.3) Depressive Symptoms—Women
Age	−0.26 * (0.12)	−0.36 ** (0.12)	−0.19 (0.23)	−0.21 * (0.10)	−0.27 ** (0.09)	−0.32 ^+^ (0.19)	−0.22 ^+^ (0.12)	−0.33 ** (0.12)	−0.19 (0.20)
Marital status (ref.: married, living together with spouse)	0.46 (2.42)	5.22 *** (1.30)	−2.61 * (1.07)	0.48 (2.55)	5.80 *** (1.17)	−3.77 *** (0.64)	0.65 (2.53)	5.70 *** (1.38)	−3.10 ** (1.06)
Self-rated health (from 1 = very good to 5 = very bad)	0.07 (1.06)	3.02 ** (1.03)	−1.53 (1.08)	0.10 (1.12)	2.94 ** (1.05)	−0.44 (1.13)	0.02 (1.07)	3.07 ** (1.03)	−1.15 (1.12)
Physical functioning (from 0 = very low to 100 = very high)	−0.08 (0.06)	0.06 (0.09)	−0.18 * (0.07)	−0.08 (0.07)	0.10 (0.08)	−0.21 ** (0.08)	−0.08 (0.07)	0.08 (0.08)	−0.18 * (0.07)
Number of important people in regular contact	0.14 (0.23)	−0.15 (0.16)	0.72 * (0.36)	0.15 (0.25)	−0.08 (0.19)	0.85 ** (0.29)	0.17 (0.24)	−0.11 (0.18)	0.79 * (0.36)
Physical job demands (from 1 = lowest decile, 10 = highest decile)	−0.33 (0.28)	−0.36 (0.25)	0.57 (1.00)						
Psycho-social job demands (1 = lowest decile, 10 = highest decile)				0.12 (0.26)	−0.06 (0.20)	1.09 ** (0.38)			
Overall job demands (1 = lowest decile, 10 = highest decile)							−0.07 (0.26)	−0.25 (0.19)	0.83 (0.72)
Constant	27.49 * (11.14)	7.09 (11.30)	33.52 ** (12.99)	22.15 * (9.98)	−2.92 (9.74)	37.33 *** (9.01)	23.21 ^+^ (11.12)	2.50 (10.37)	30.97 ** (10.53)
Number of individuals	3186	1661	1525	3186	1661	1525	3186	1661	1525
Number of observations	3229	1688	1541	3229	1688	1541	3229	1688	1541
R^2^	0.12	0.31	0.43	0.11	0.27	0.57	0.11	0.29	0.48

Unstandardized beta-coefficients are reported; cluster-robust standard errors in parentheses. *** *p* < 0.001, ** *p* < 0.01, * *p* < 0.05, ^+^ *p* < 0.10.

**Table 3:** Determinants of depressive symptoms (total sample and stratified by sex)—*with objective job demands of one’s partner* (1: physical job demands, 2: psycho-social job demands; 3: overall job demands) as main independent variable. Results of multiple linear FE regressions.

	(1.1) Depressive Symptoms—Total Sample	(1.2) Depressive Symptoms—Men	(1.3) Depressive Symptoms—Women	(2.1) Depressive Symptoms—Total Sample	(2.2) Depressive Symptoms—Men	(2.3) Depressive Symptoms—Women	(3.1) Depressive Symptoms—Total Sample	(3.2) Depressive Symptoms—Men	(3.3) Depressive Symptoms—Women
Age	−0.27 ** (0.09)	−0.27 * (0.12)	−0.31 ** (0.12)	−0.27 ** (0.09)	−0.27 * (0.12)	−0.29 * (0.13)	−0.27 ** (0.09)	−0.27 * (0.12)	−0.31 * (0.12)
Marital status (ref. married, living together with spouse)	2.87 (2.58)	1.83 (1.61)	3.85 (3.41)	2.16 (2.91)	2.03 (1.65)	1.84 (5.18)	2.62 (2.75)	1.97 (1.63)	3.69 (4.28)
Subjective health (1 = very good, 5 = very bad)	2.42 ** (0.77)	1.37 (0.98)	3.47 *** (0.93)	2.39 ** (0.80)	1.31 (0.98)	3.16 ** (1.09)	2.41 ** (0.78)	1.34 (0.99)	3.38 *** (0.09)
Physical functioning (0 = very low, 100 = very high)	−0.12 *** (0.03)	−0.12 (0.09)	−0.12 ** (0.04)	−0.11 *** (0.03)	−0.13 (0.09)	−0.11 ** (0.04)	−0.11 *** (0.03)	−0.12 (0.09)	−0.11 ** (0.03)
Number of important people in regular contact	0.30 * (0.14)	0.11 (0.17)	0.45 ^+^ (0.24)	0.28 * (0.14)	0.11 (0.17)	0.45 ^+^ (0.24)	0.30 * (0.14)	0.11 (0.17)	0.46 ^+^ (0.24)
Physical job demands (1 = lowest decile, 10 = highest decile)	0.61 ^+^ (0.31)	−0.29 (0.32)	1.48 *** (0.38)						
Psycho-social job demands (1 = lowest decile, 10 = highest decile)				−0.02 (0.16)	−0.06 (0.21)	0.00 (0.24)			
Overall job demands (1 = lowest decile, 10 = highest decile)							0.35 (0.24)	−0.18 (0.23)	0.94 ** (0.36)
Constant	17.41 ^+^ (7.14)	26.32 ^+^ (14.28)	10.45 (8.44)	20.69 ** (7.63)	25.71 ^+^ (14.21)	19.21 ^+^ (10.28)	18.32 * (7.30)	26.05 ^+^ (14.28)	12.48 (9.24)
Number of individuals	2518	1267	1251	2518	1267	1251	2518	1267	1251
Number of observations	2669	1334	1335	2669	1334	1335	2669	1334	1335
R^2^	0.24	0.20	0.35	0.21	0.19	0.24	0.22	0.19	0.29

Unstandardized beta-coefficients are reported; cluster-robust standard errors in parentheses. *** *p* < 0.001, ** *p* < 0.01, * *p* < 0.05, ^+^ *p* < 0.10.

## Data Availability

The data used in this study are third-party data. The anonymized data sets of the DEAS (1996, 2002, 2008, 2011, 2014, 2017, and 2020) are available for secondary analysis. The data has been made available to scientists at universities and research institutes exclusively for scientific purposes. The use of data is subject to written data protection agreements. Microdata of the German Ageing Survey (DEAS) are available free of charge to scientific researchers for non-profitable purposes. The FDZ-DZA provides access and support to scholars interested in using DEAS for their research. However, for reasons of data protection, signing a data distribution contract is required before data can be obtained. For further information on the data distribution contract, please see https://www.dza.de/en/research/fdz/access-to-data/formular-deas-en-english (accessed on 3 October 2021).
